# Pathophysiology, Diagnosis and Treatment of Spontaneous Coronary Artery Dissection in Peripartum Women

**DOI:** 10.3390/jcm11226657

**Published:** 2022-11-10

**Authors:** Marta Cano-Castellote, Diego Fernando Afanador-Restrepo, Jhonatan González-Santamaría, Carlos Rodríguez-López, Yolanda Castellote-Caballero, Fidel Hita-Contreras, María del Carmen Carcelén-Fraile, Agustín Aibar-Almazán

**Affiliations:** 1 Department of Health Sciences, Faculty of Health Sciences, University of Jaén, 23071 Jaén, Spain; 2 Faculty of Distance and Virtual Education, Antonio José Camacho University Institution, Santiago de Cali 760016, Colombia; 3 ZIPATEFI Research Group, Faculty of Health Sciences and Sports, University Foundation of the Área Andina, Pereira 660001, Colombia; 4 Faculty of Health Sciences, Technological University of Pereira, Pereira 660001, Colombia; 5 Nutrition Sciences Postgraduate, Faculty of Nutrition Sciences, University of Sinaloa, Culiacan 80019, Mexico; 6 Lecturer University Schools Gimbernat, University of Cantabria, 39005 Santander, Spain

**Keywords:** SCAD, pregnancy, postpartum period, disease management

## Abstract

Spontaneous coronary artery dissection (SCAD) is an infrequent cause of nonobstructive ischemic heart disease in previously healthy young women and therefore is not usually considered in differential diagnoses. The overall incidence of SCAD in angiographic series is between 0.28 and 1.1%, with a clear predominance in young, healthy women (70%) of whom approximately 30% are in the postpartum period. In the United Kingdom, between 2008 and 2012, SCAD was the cause of 27% of acute myocardial infarctions during pregnancy, with a prevalence of 1.81 per 100,000 pregnancies. Regarding the mechanism of arterial obstruction, this may be due to the appearance of an intramural hematoma or to a tear in the intima of the arteries, both spontaneously. Although multiple diagnostic methods are available, it is suggested to include an appropriate anamnesis, an electrocardiogram in the first 10 min after admission to the service or the onset of symptoms, and subsequently, a CT angiography of the coronary arteries or urgent coronary angiography if the hemodynamic status of the patient allows it. Treatment should be individualized for each case; however, the appropriate approach is generally based on two fundamental pillars: conservative medical treatment with antiplatelet agents, beta-blockers, and nitrates, and invasive treatment with percutaneous coronary intervention for stent implantation or balloon angioplasty, if necessary.

## 1. Introduction

Several pathologies affect the function and structure of the heart; the most common is coronary artery disease [1,2]. Any situation that decreases blood flow through the coronary arteries makes the heart susceptible to myocardial ischemia [3]. Today, the most common cause of ischemic heart disease is coronary arteriosclerosis, but many other diseases, perhaps less well known, both congenital and acquired, are also found that affect the coronary arteries from anomalous coronary origin, from stenosis or high location of the ostium to hypoplasia of the coronary arteries, dissection, myocardial bridging, aneurysm, or vasculitis [4].

The prevalence of ischemic heart disease (IHD) in the general population is very high; in the United States, every year more than eight million inhabitants present symptoms related to IHD and more than 700,000 are diagnosed with the pathology [5]. It is estimated that 42% of all deaths related to cardiovascular diseases are due to ischemic heart disease [6]. Different authors have established over time important differences in the prevalence of IHD between men and women, such as in the case of the original Framingham Study cohort, from whom a follow-up of 44 years managed to determine that the risk of developing IHD in men was 49% and in women 32% when they were younger than 40 years of age [7,8]; even though being male is considered a risk factor for IHD, the reason for this risk is still unclear [9]. However, this does not discount the presence of cardiovascular diseases in women; in Spain during 2018, 64,897 women died from these diseases, 20% (12,729) of these deaths due to coronary heart disease [10].

Spontaneous coronary artery dissection (SCAD), according to the European Society of Cardiology, is classified as a Myocardial Infarction with Non-Obstructive Heart Disease (MINOCA), due to the fact that this pathology usually presents as a non-obstructive stenosis >50% [11,12,13]. However, this pathology is one of the less frequent causes of MINOCA, which is part of the Acute Coronary Syndromes framed in the IHD [14,15]. The first description of SCAD in the literature comes from Harold Pretty in 1931, as an autopsy finding in a 42-year-old woman who died after presenting chest pain [16,17,18]. Most of the published evidence on this pathology during the succeeding decades was reduced to a few clinical cases or small case series [19,20,21,22]. Nonetheless, in 2022 a large-scale study took place, observing retrospectively over 22 years 75 women who presented SCAD during peripartum [23].

Due to its rarity, and the fact that multiple pathologies share its symptomatology, such as gastroesophageal reflux and musculoskeletal pain, SCAD is a difficult disease to diagnose during pregnancy [24]. Spontaneous coronary artery dissection predominantly affects previously healthy young women, a population that, even with compatible semiology, would not raise suspicion of MINOCA as part of the initial differential diagnosis, which has the repercussions of delayed treatment and the lives of these women being put at risk [25].

Given the slowdown in the rate of decline of cardiovascular mortality that has been observed in recent years [10], it is important to produce new and updated evidence regarding the different cardiovascular diseases. Therefore, this review aims to update the evidence on SCAD, its pathophysiology, diagnosis, and treatment.

## 2. Epidemiology

Spontaneous coronary artery dissection without atherosclerosis is a rare finding [26]; however, debate rages in the United States and Canada as to whether SCAD should continue to be considered a rare condition [27,28]. Spontaneous coronary artery dissection is an uncommon cause of Sudden Cardiac Arrest (SCA) in the general population, but its prevalence has increased in women during the puerperium [27]. The overall incidence of SCAD in angiographic series is between 0.28 and 1.1%, with a clear predominance in young, healthy women (70%) of whom approximately 30% are in the postpartum period (10% in late pregnancy and 20% postpartum) [4,29]. Other studies have reported that SCAD was the cause of 27% of acute myocardial infarctions occurring during pregnancy [30,31], with a prevalence of 1.81 per 100,000 pregnancies, with 69.9% of cases occurring during the puerperium [24]. Additionally, evidence suggests that SCAD mainly affects middle-aged women, between 44 and 56 years [16,30,32,33,34,35,36,37]. Likewise, a suspicion arises regarding race; although most of the women who presented with SCAD were Caucasian, no significant differences have been found to confirm this assumption [38].

An important feature to highlight about the SCAD population is the tendency to have lower, although not absent, rates of cardiovascular risk factors such as diabetes, smoking, and hyperlipidemia compared to the population suffering from acute atherosclerotic myocardial infarction and the United States age-matched average [39,40]. However, evidence shows that in the case of hypertension, patients with SCAD tend to be on par with the age-matched average [41].

Regarding the most frequently affected artery in SCAD, in more than half of the cases (44–80%) the anterior descending branch of the left coronary artery is damaged, followed by the right coronary artery, the common coronary trunk, and finally the circumflex branch of the left coronary artery [16,42]. Simultaneous multivessel involvement is the most unusual form of presentation in SCAD but may occur in up to 19% of patients [37].

## 3. Pathophysiology

Spontaneous coronary artery dissection is a pathological phenomenon that occurs when the arterial wall layers separate with or without an associated intimal tear, creating a false lumen. Usually the dissection appears 2 cm from the ostium of the coronary arteries and extends distally, between the intimal and medial layers, or between the media and the adventitia [43]. Several authors have attempted to describe the mechanism of injury of this pathology; however, this has not yet been elucidated [43]. On the one hand it is believed that SCAD is initiated by a tear in the intima that results in a dissection that spreads through the tunica media, while other authors claim that this pathology is produced by a dissecting hematoma in the tunica media, generated by the rupture of the vasa vasorum [44] or by mechanical damage to the arterial wall, followed by an inflammatory reaction [45]. Besides, evidence shows that the false lumen created by the tear or intraparietal hematoma compresses the true lumen and can compromise arterial flow, generating a complete or partial obstruction resulting in myocardial ischemia [4,42].

This condition is more common during pregnancy, due to the significant changes that occur during this period, which have hemodynamic [46], respiratory, and metabolic [47] responses that can last for 6 months after delivery [35,42,48]. This is caused by a significant increase in blood volume due to pregnancy, which places the cardiovascular system under high levels of stress [49]. As a result, myocytes change their elastic and contractile capacity [46], while the respiratory system increases oxygen consumption [50,51] to respond to the new demands [52].

The physiological stress generated by pregnancy can cause the vascular endothelium, extracellular matrix, and epicardial fat to be involved in pathological processes [53,54] to which myocytes, due to their high presence [55] and function in the heart [56,57] are not exempt. The stress produced by pregnancy causes changes in myocyte ultrastructure followed by an increase in the size of the heart in response to the mechanical and growth stimuli induced during gestation [58]. Pregnancy-induced cardiac hypertrophy is a necessary physiological situation of reversible nature [59], which can lead to a pathological process that is no longer reversible where abnormalities in tissue and cell architecture occur and the ability of cardiac tissue to restore itself is reduced [60,61,62].

Regarding the endothelium, the actions of the endothelial nitric oxide synthase (eNOS) and eNOS-independent pathways must ensure sufficient nitric oxide production to maintain vascular tone [63,64,65]. Since the endothelium is in direct contact with the blood, it is sensitive to hemodynamic changes [66,67]. Under non-pathological conditions, the endothelial layer adapts to the hemodynamic demands of pregnancy, thus ensuring uniform blood flow [68]. However, when major alterations such as turbulent blood flow, decreased vasomotor tone, and shear stresses occur, among others, endothelial dysfunction is generated [69]. Most of the factors that cause damage to the cardiovascular system are related to endothelial dysfunction [70]. The rate of endothelial cell turnover is low regardless of the shear stress and the level of damage that may be generated by cytotoxic agents [71]. Although mechanical endothelial rupture is often associated with chronic cardiovascular disease, sudden large endothelial rupture has been reported in the past [72]. During pregnancy, plasma volume expansion leads to changes in the vascular endothelial growth factor, placental growth factor, and soluble growth factor receptors, which eventually dilute in the blood [73] affecting endothelial cell permeability and architecture [74].

Another possible cause of this pathology in pregnant women is attributed to the hormonal changes that occur during this period [75,76,77]. The increase in estrogens generates changes in the proteins and mucopolysaccharides found in the tunica media of the arteries, favoring the breakdown of collagen and elastin, and the reduction of proteoglycans leading to a thinning of the coronary wall [35,42,75]. Increased progesterone resulting from increased hemodynamic stress [78,79] can cause hyperplasia and the hypertrophy of smooth muscle fibers, weakening the arterial tunica media. The cumulative effect of these changes in successive pregnancies is why multiparity is considered a risk factor for SCAD [80].

Finally, pre-existing diseases that may favor SCAD include chest trauma, the use of oral contraceptives and cyclosporine, abnormalities of the coronary tree, connective tissue diseases such as Marfan syndrome or Ehlers-Danlos disease type IV, vasculitis such as Kawasaki disease, hypereosinophilic syndrome, ulcerative colitis, and systemic lupus erythematosus [4,36,81]. Additionally, other at risk groups for the development of SCAD are elderly pregnant patients, smokers, multiparous patients, or cocaine users [82,83].

## 4. Clinical Presentation

The clinical presentation of SCAD covers a wide spectrum of signs and symptoms [29,42,84,85,86]. Initially, the patient with SCAD may present with no symptoms; however, the most common symptomatology is the one typically associated with acute myocardial infarction with ST segment elevation, respiratory distress [87], or directly sudden death, the latter being more frequent in patients with left coronary artery involvement [36,88]. Additionally, some reports of SCAD have been accompanied by unstable angina, but this is extremely rare [48,89,90]. Symptomatology usually begins after delivery during the puerperium [91,92,93], between day 3 and day 210 [87]; however, cases have been reported during the second [94,95] and third trimester [96,97,98].

Despite the wide variety of symptoms, all authors agree that the most common manifestation is chest pain [99,100,101], which is quite nonspecific, since the clinical symptom par excellence of myocardial ischemia of any origin is precordial pain [102,103]. This usually has an acute, sudden onset, with oppressive or stabbing, anginal, substernal, retrosternal, or interscapular characteristics [104,105,106]. Along with chest pain, some patients report dyspnea and diaphoresis, with nausea and profuse sweating [107]. The pain is usually of high intensity and sometimes radiates to the left arm or to the neck and jaw in the form of paresthesia, dysesthesia, or pressure, and its duration before consulting the emergency department varies in different publications, being reduced to a period of time between 1 h and 2–3 weeks, depending on the intensity [82].

Additionally, although it does not always occur, SCAD can present with hypotensive hemodynamic alterations, bradycardia, and/or ventricular fibrillation [107]. Moreover, although AMI usually occurs with an ST elevation evaluated by ECG, evidence exists of patients with SCAD in whom the AMI occurred without ST elevation; therefore, this type of AMI could alert clinicians to a possible SCAD [82].

Finally, the recurrence of SCAD has been described by different authors; however, a great discordance exists in the cases observed. Recurrence rates range between 17 and 50%, being more frequent between 30 and 60 days after the first episode [16,37,42,108,109].

## 5. Diagnosis

**Figure 1:**
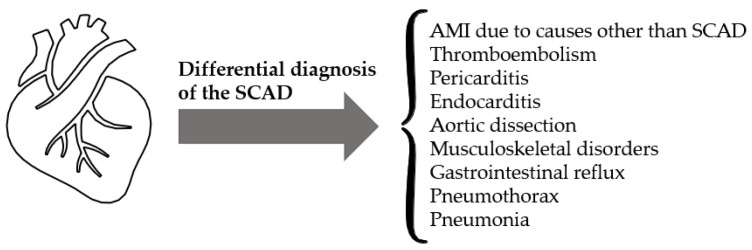
Differential diagnosis of Spontaneous Coronary Artery Dissection.

During pregnancy and the postpartum period, cardiovascular emergencies are rare but potentially fatal; therefore, to properly diagnose SCAD it is important to perform an exhaustive differential diagnosis in which pathologies such as AMI due to other causes must be ruled out, as well as pulmonary thromboembolism, pericarditis, endocarditis, aortic dissection, musculoskeletal disorders, gastrointestinal reflux, pneumothorax, pneumonia, or cardiac tamponade, among others (Figure 1) [29,82].

Acute myocardial infarction is the most common cause of cardiac emergency in pregnant or puerperal women, and among the possible causes of AMI stands out SCAD, producing about 23–35% of all AMI in women under 50 years of age, and 40% in pregnant or puerperal women [82,110]. The first angiographic diagnosis was made in 1978, and until then most cases were diagnosed by autopsy [4,30], since in young women without coronary risk factors it did not seem likely that SCAD was the cause of their chest pain [27,86,111]. At present, following different studies, evidence has shown that diagnostic tests such as ECG, CT-scan, MRI, or coronary angiogram should be used in conjunction with angiography to obtain a more accurate diagnosis of SCAD [112,113,114,115].

The 12-lead ECG is described as the first diagnostic test to be performed, which should be done within 10 min of triage if the patient manifests chest pain in the emergency department, or at the onset of chest pain if the patient is already admitted [82]. Electrocardiographic changes are variable, depending on the affected artery and the severity of the myocardial ischemia, so we can find a multitude of alterations [116,117]. In 75–80% of patients with SCAD, the ECG shows STEMI [42,82]. However, other possible ECG alterations associated with SCAD exist, such as a pattern with bigeminy and no ST segment or T-wave changes [4,118] or diffuse ST segment depression [29].

On the other hand, cardiac biomarkers have also been useful for the early diagnosis of SCAD [109]; these include troponins, CK, CK-mb, and atrial natriuretic peptide [82]. Evidence suggests that even if cardiac biomarkers are within the normal range at the beginning of the diagnostic process, constant monitoring should be taken into account because, when SCAD occurs, the values do not change initially, but after a few hours they increase. Such was the case reported by Jofré et al. [29], where at the beginning troponin levels were at 0.45 ng/mL, but after some time these rose to over 50 ng/ml, the initial CK of 72 U/L increased to 3800 U/L, and an initial CK-mb of 2.8 U/L increased to >300 U/L.

Another diagnostic test that is easy to perform noninvasively and has been reported to be very useful is transthoracic echocardiography [119,120]. Different authors have made important clinical findings that they presented in different case studies; Shahzad et al. [4] described a case of SCAD with an LVEF of 30–35% and LV hypokinesia, while Petrou et al. [48] showed a patient with global hypokinesia and an LVEF of 25%. Echocardiography has been useful to evaluate cardiac function and structure, finding an association between partial or global myocardial wall hypokinesia and a left ventricular ejection fraction below 50% with SCAD; however, these findings are not entirely specific.

Emergent coronary angiography is usually the best diagnostic technique to identify SCAD [121] and was considered the gold standard test [109,122]. This diagnostic test should be performed whenever signs of acute myocardial infarction are present in the ECG [82,123]. The typical appearance of SCAD in coronary angiography is an image of a radiolucent intimal tear or spiral dissection creating a false lumen, with obliteration of the true lumen of the coronary artery, with or without intramural hematoma, and with delayed contrast clearance [30,124,125]. Coronary angiography is able to diagnose SCAD in addition to differentiating the affected artery; however, this is not always shown clearly so other findings must be taken into account for its diagnosis such as a narrowing of the arterial lumen or its total occlusion by an intramural hematoma [36]. Ito et al. [14] described that, in cases of diagnostic doubt due to the narrowing of the coronary arterial lumen without obvious findings of SCAD, it is advisable to inject intracoronary nitroglycerin to rule out a possible vasospasm.

Regarding the radiation to which the fetus is exposed during diagnostic coronary angiography, the evidence suggests that the fetal radiation dose is very low (0.074 mGy); according to the American College of Obstetricians and Gynecologists, exposure to less than 50 mGy of radiation does not produce any adverse obstetric or fetal effects [81,126]. However, it is important to note that coronary angiography is a potentially harmful procedure that can lead to increased coronary artery dissection [127].

Moreover, other diagnostic methods have been used in SCAD and are recommended prior to coronary angiography, such as optical coherence tomography and CT angiography [128,129]. Optical coherence tomography is a diagnostic test that allows the observation of an intravascular image of the artery and its wall, determining the presence of a double lumen, intimal tear, or circumferential or longitudinal dissection, including the existence or absence of an associated thrombus [130,131]. However, as this is an invasive procedure, it presents the same risk as coronary angiography of worsening arterial dissection [132]. Concerning CT angiography, it is indicated, when the patient is stable and coronary angiography can be postponed or estimated, that it may not be necessary [82]. Computerized tomography angiography is a noninvasive method that allows the detection of features such as the typical image of an intimal flap with opacification and the late contrast washout in the false lumen, or a decrease in the lumen of the vessel that may simulate an intracoronary thrombus or stenosing atherosclerosis, compatible with the presence of an intramural hematoma [128,133,134].

**Figure 2:**
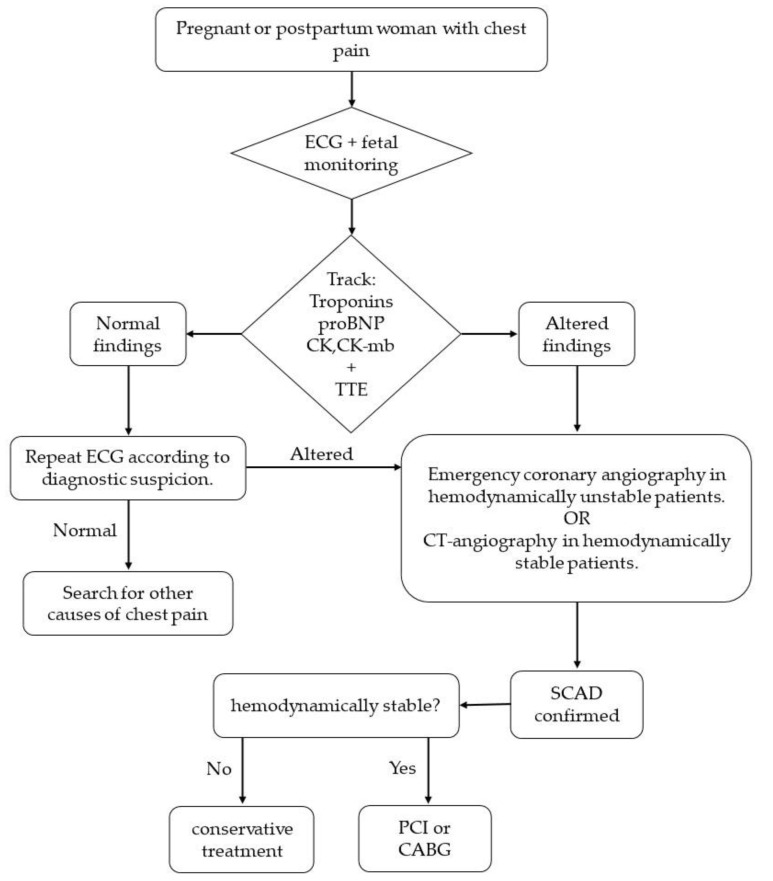
Suggested protocol for the diagnosis and treatment of SCAD. ECG: electrocardiogram; TTE: transthoracic echocardiogram; PCI: percutaneous coronary intervention; CABG: coronary artery bypass graft.

The diagnosis of SCAD is complex; therefore, multiple tests and proper clinical judgment are necessary. A protocol for the diagnosis and treatment of SCAD is proposed in Figure 2.

## 6. Treatment

The management of a patient with SCAD depends fundamentally on the clinical presentation, the hemodynamic compromise, the size and site of the dissection, and the number of affected vessels, which means that it must be individualized for each patient [4]. The evidence agrees that the treatment of SCAD should be carried out by a multidisciplinary team composed of cardiologists, intensivists, and gynecologists, aiming primarily at the rapid stabilization of the patient, followed by continuous hemodynamic monitoring [109]. The success of the treatment depends directly on the individualization of the patient and the time of diagnosis, and it is possible to observe a decrease in the mortality rate as the diagnostic process improves thanks to the development of new technologies. Between 1922 and 1995 the mortality rate for SCAD was close to 20%, while by 2012 it was around 10% [30]; finally, in the last decade it has become less than 5% [135].

### 6.1. Conservative Treatment

In patients with a stable hemodynamic status or small vessel involvement, conservative treatment or medical treatment is indicated. In some case reports, this is the most commonly used treatment [16]. Initially, this treatment consisted of the application of fibrinolytics in the acute phase [4,30,42,48], drugs that have been shown to be effective in AMI with ST elevation [136]. However, it was later demonstrated that they favor bleeding and may increase intraparietal hematoma, enlarging the compression of the true arterial lumen, and worsening the clinical course [125,137]. Currently, numerous studies have ruled that an antiplatelet drug (clopidogrel or aspirin) should be used in association with beta-blockers, nitrates, and angiotensin-converting enzyme inhibitors (ACE inhibitors). In cases in which SCAD occurred immediately after delivery, intravenous oxytocin or misoprostol was administered to prevent hypotension [138]. Table 1 summarizes the different recommended and non-recommended pharmacologic strategies used in the management of SCAD.

### 6.2. Invasive Treatment

In patients with unstable hemodynamics or active ischemia, especially when the proximal third of a vessel has been severely affected, percutaneous coronary intervention or coronary artery bypass grafting of the coronary artery is the most appropriate option [93,145]. Percutaneous coronary intervention permits the correction of the arterial portion with reduced caliber by implanting a stent or dilating this area with a balloon angioplasty. The use of stenting has proved to be effective, restoring flow in the true lumen, reducing ischemia, and sealing the dissection [48]. The success rate reported in small case reviews ranges between 47% and 91% [145]; however, other authors have reported a complication rate in between 20% and 60% of cases [82,108]; this is because the arteries affected by SCAD are more susceptible to the generation of iatrogenic reactions during catheter manipulation, angioplasty, or stenting, which could result in an anterograde or retrograde extension of the initial dissection [146]. Although several methods exist that attempt to mitigate the adverse effects of percutaneous coronary intervention, such as the use of longer stents that prevent stent migration or the propagation of the dissection, or the use of angioplasty balloons with the capacity to modify the plaque and depressurize the false lumen, insufficient evidence exists yet to support them [37].

When the case is severe and multivessel or left main artery common trunk alteration is evident, coronary artery bypass grafting is necessary [147,148]. This intervention is also usually performed when percutaneous coronary intervention has failed [123,149]. The evidence surrounding CABG is limited to case studies, case series, or retrospective studies, so its long-term effects have not yet been fully determined; however, short-term results tend to be favorable, although the risk of bypass graft occlusion is high [150].

Other interventions used for the treatment of SCAD include cardiocirculatory support in the acute phase. Aortic balloon counterpulsation is used to optimize cardiac output, increase coronary blood flow, and maximize perfusion at the level of the uterus and myocardium [151]; however, its use in SCAD remains limited. The utilization of aortic balloon counterpulsation, left ventricular assist, or an artificial heart as possible alternatives to cardiac transplantation are indicated strategies when myocardial revascularization fails with left ventricular dysfunction [77,152,153]. The use of extracorporeal membrane oxygenation as a life-sustaining measure during cardiogenic shock triggered by SCAD has ample evidence; when medical or surgical treatment fails, the application of this intervention improves mortality rates especially in young patients without other concomitant pathologies and in potentially reversible situations [154,155].

## 7. Conclusions

Spontaneous coronary artery dissection is a complex disease, with a sudden onset that can lead to many complications. Given the fact that it usually occurs in young pregnant women with a lack of cardiovascular risk factors, and the similarity of its signs and symptoms to other more prevalent diseases, it is difficult to diagnose.

Currently, the best way to diagnose SCAD is through coronary angiography; however, this technique involves different risks, including worsening coronary dissection; therefore, electrocardiography, the tracking of cardiac biomarkers such as troponin, CK and CK-mb, and transthoracic echocardiography are considered adequate techniques for diagnosis when it is not desirable to take these risks. Regarding treatment, as long as the hemodynamic situation of the patient allows it, the use of drugs such as aspirin plus Clopidogrel is the best option; if this is not sufficient, the invasive treatments with the most evidence are percutaneous coronary intervention or coronary artery bypass grafting.

## Figures and Tables

**Table 1:** Use of drugs in the treatment of SCAD.

Drugs Used in the Conservative Treatment			
DRUGS	USE	ADVANTAGES	DISADVANTAGES
ASPIRIN	First-line treatment. Most-used drug for acute and long-term treatment of SCAD.	Antiaggregant. Low hemorrhagic risk and proven benefit in patients with ACS and in secondary prevention of coronary artery disease [139,140].	Low hemorrhagic risk [140].
BETA- BLOCKERS	First-line treatment.	Reduce arterial wall stress [141].	Avoid in patients with severe asthma or COPD [141].
NITRATES	First-line treatment.	Vasodilator [142].	
ACE Inhibitors	They are not a first-line treatment.	Indicated only in patients with a significant decrease in LVEF after ACS (EF < 40%) [142].	
ANTI COAGULANTS (LMWH)	Controversial use; initially administered in patients with ACS.	Not recommended [107].	They have been shown to increase the risk of dissection spread and intramural hematoma [82].
FIBRINOLYTICS.	Not recommended.	Not recommended [107].	They have been shown to promote bleeding and increase intraparietal hematoma, enlarge compression of the true arterial lumen, and worsen the clinical picture [107].
Drugs used in the Invasive treatment			
ASPIRIN + CLOPIDOGREL	First-line treatment.	Dual antiplatelet therapy may be beneficial in reducing the false lumen thrombus created for SCAD, thereby reducing the decrease in true lumen caliber [143].	Increased risk of bleeding [144].

LMWH: Low-molecular-weight heparins; SCAD: Spontaneous Coronary Artery Dissection; ACS: Acute Coronary Syndrome; LVEF: left ventricular ejection fraction; EF: Ejection Fraction; COPD: Chronic obstructive pulmonary disease.

## Data Availability

Not applicable.
